# Proper evaluation of chemical cross-linking-based spatial restraints improves the precision of modeling homo-oligomeric protein complexes

**DOI:** 10.1186/s12859-019-3032-x

**Published:** 2019-09-09

**Authors:** Aljaž Gaber, Gregor Gunčar, Miha Pavšič

**Affiliations:** Department of Chemistry and Biochemistry, Faculty of Chemistry and Chemical Technology, University of Ljubljana, Večna pot 113, 1000 Ljubljana, SI Slovenia

**Keywords:** Chemical cross-linking, Homo-oligomers, Cross-link assignation ambiguity, Solvent accessible surface distances, Modeling

## Abstract

**Background:**

The function of oligomeric proteins is inherently linked to their quaternary structure. In the absence of high-resolution data, low-resolution information in the form of spatial restraints can significantly contribute to the precision and accuracy of structural models obtained using computational approaches. To obtain such restraints, chemical cross-linking coupled with mass spectrometry (XL-MS) is commonly used. However, the use of XL-MS in the modeling of protein complexes comprised of identical subunits (homo-oligomers) is often hindered by the inherent ambiguity of *intra*- and *inter*-subunit connection assignment.

**Results:**

We present a comprehensive evaluation of (1) different methods for *inter*-residue distance calculations, and (2) different approaches for the scoring of spatial restraints. Our results show that using Solvent Accessible Surface distances (SASDs) instead of Euclidean distances (EUCs) greatly reduces the assignation ambiguity and delivers better modeling precision. Furthermore, ambiguous connections should be considered as *inter*-subunit only when the *intra*-subunit alternative exceeds the distance threshold. Modeling performance can also be improved if symmetry, characteristic for most homo-oligomers, is explicitly defined in the scoring function.

**Conclusions:**

Our findings provide guidelines for proper evaluation of chemical cross-linking-based spatial restraints in modeling homo-oligomeric protein complexes, which could facilitate structural characterization of this important group of proteins.

**Electronic supplementary material:**

The online version of this article (10.1186/s12859-019-3032-x) contains supplementary material, which is available to authorized users.

## Background

To understand the mechanisms of how proteins drive and regulate biological processes, detailed knowledge of their structure is fundamental. Obtaining structural information is a long and complicated process, even more so when the object of investigation is a complex of multiple proteins. Despite advances in methods for high-resolution structure determination, structural characterization of protein complexes often relies on computational algorithms for protein-protein docking that are guided by spatial restraints obtained from experimental data [1]. Such information, albeit being of low resolution, substantially improves docking accuracy and precision [2]. This approach is also often employed to model the quaternary structure of protein complexes comprised of multiple identical subunits (homo-oligomers), and can be successful when the high-resolution structure of a subunit is available. This is important because 30–50% of all proteins can self-associate to form such multi-subunit complexes [3]. Here, spatial restraint-driven docking not only helps in the determination of the quaternary structure of protein complexes but can also provide valuable insight into the mechanism of protein self-association [4, 5].

Among the most frequently used spatial restraints are those acquired from chemical cross-linking, coupled with mass spectrometry (XL-MS) [6–8]. Cross-linker, a bi-reactive chemical component, connects specific amino acid residues located at an appropriate distance in the 3D structure of the protein complex. The length of the cross-linker defines the maximal distance between the reactive ends of the cross-linked residues. This length, combined with the length of the side chains of connected residues and increased for a factor accounting for the protein’s backbone flexibility, is used to determine the upper limit of the distance between the C_α_ atoms (C_α_-C_α_ distance) of the cross-linked residues [9]. In the distance restraint-guided docking, multiple models of complexes are generated and used to calculate *inter*-residue distances of experimentally identified cross-links. To confirm or reject individual models, distances calculated from these models are compared with the theoretical upper limit distances; if a model-derived distance fits the theoretically possible distance limit, the model could be considered as probable.

However, modeling of homo-oligomeric complexes based on XL-MS spatial restraints involves a challenge that is absent from heterogenous complexes, namely all interacting subunits have the same amino acid sequence. As sequences of the connected peptides are derived from their mass spectra, and identification of the cross-linked residues is in turn sequence-based, *intra-* and *inter-*subunit connections are therefore inherently ambiguous. Here, each identified connection can be either a result of *intra-* or *inter*-subunit connection or both. In homo-dimers, this problem can be circumvented by labeling a single subunit with heavy isotopes during protein expression [10–13], and comparative mass spectrometry approaches have been developed to distinguish between *intra*- and *inter*-subunit connections based on the abundance of individual cross-links [14–16]. However, both approaches have their limitations and do not apply to all investigations.

**Fig. 1:**
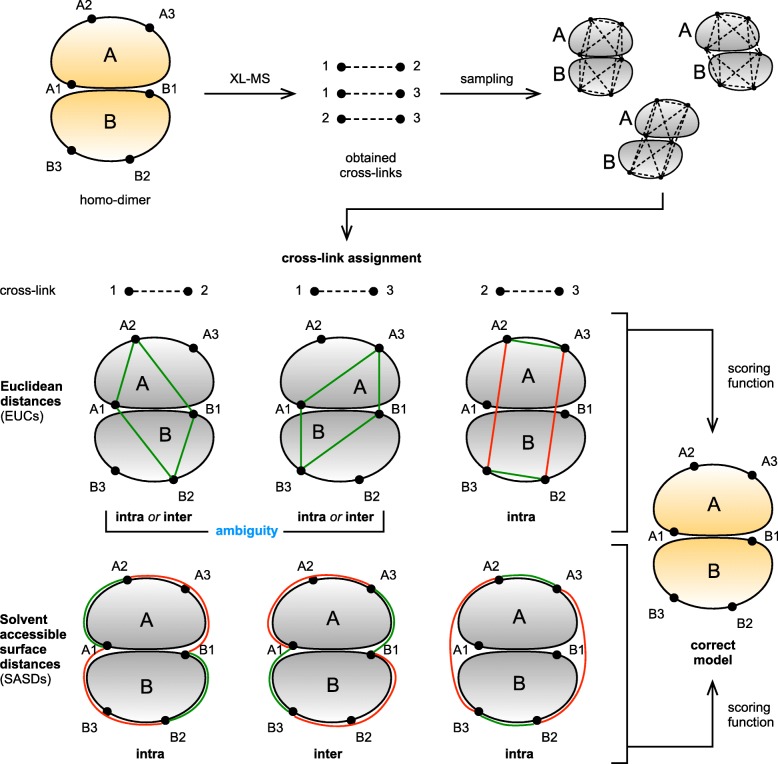
Schematic representation of EUCs and SASDs and their ability to resolve the ambiguity of cross-linker assignation. Here, a simplified example based on a homo-dimer is shown where cross-linking results in three cross-links (1–2, 1–3, and 2–3). During sampling, multiple homo-dimer models can be generated with different relative orientation between the two subunits (only three models are shown). The ability to correctly classify cross-links as inter- or intra-subunit, combined with an appropriate scoring function, helps in generating the correct model of the homo-oligomer

To better understand the ambiguity problem and its possible solutions, we investigated how *inter*-residue distance in combination with different computational approaches can help to resolve the cross-link assignation ambiguity. Two aspects were considered: (1) methods for *inter*-residue distance calculations, and (2) scoring approaches for proper evaluation of ambiguous cross-links as spatial restraints. We performed our investigation by analyzing available high-resolution structures of homo-oligomers. Here we calculated their *inter*-residue distances using two different methods and compared the precision of protein-protein docking predictions aimed to recreate initial structures by using simulated cross-linking data together with different scoring approaches. For the first part, we analyzed whether using Solvent Accessible Surface Distances (SASDs) better discriminates between *intra*- and *inter*-subunit connections than most commonly used Euclidean distances (EUCs). While SASDs account for residue solvent accessibility and also for space occupied by proteins, EUCs completely disregard both which can lead to false positive assignations [8, 17, 18] (Fig. 1). In the second part, we compared the effect of different scoring approaches since currently there is no consensus on how to properly score ambiguous cross-links (or whether to use them as spatial restraints at all). Third, we examined how can symmetry, present in almost all homo-oligomers, be used as additional restraint for modeling to give the best possible outcome and also whether it can be used to resolve the ambiguity. Our results provide basic guidelines for efficient use of XL-MS data in modeling homo-oligomers.

## Results

### Solvent accessible surface distances are better at distinguishing between *intra*- and *inter*-subunit connections

Calculating SASDs instead of EUCs has been shown to improve the precision of modeling proteins and protein complexes, by more accurately defining which *inter*-residue distances agree with the experimental data [8, 18, 19]. We wanted to evaluate if calculating SASDs instead of EUCs can also help distinguish between *intra-* and *inter-*subunit cross-links in homo-oligomers. We calculated non-redundant Cα-Cα SASDs and EUCs between lysine residues in 13,110 high-resolution homo-oligomeric protein complex structures (see Materials and Methods). Connections between individual residues were then assigned, based on the corresponding *inter*-residue distances: *Intra*-subunit, *Inter*-subunit, *Ambiguous,* if both *Intra*-subunit and *Inter*-subunit alternatives were valid, or Non-accessible if the distance was too long or the endpoint residue was not accessible to the cross-linker. We used 33 Å as the upper threshold for the assignation – the distance used as a threshold for two commonly used cross-linkers disuccinimidyl suberate (DSS) and bis(sulfosuccinimidyl) suberate (BS3) [18].

**Fig. 2:**
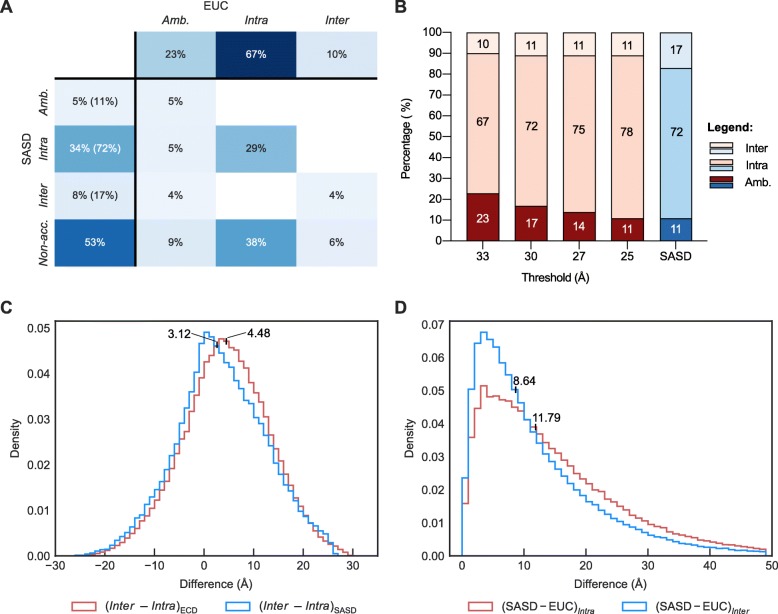
Comparison of using SASDs over EUCs to reduce the *Intra*- vs. *Inter*-subunit ambiguity based on the inter-residue distance. **a** A heatmap showing a redistribution of assignations when using SASDs over EUCs. Values represent the proportion of individual assignations. The total values obtained with each method are shown in header row and column, while the body of the heatmap shows the proportions of combinations. Values in parentheses represent the relative proportion of assignations obtained using SASDs, excluding *Non-accessible*. **b** Comparison of redistribution of assignations between different EUC thresholds (red bars) and using SASDs over EUCs (blue bars). Values inside bar sections represent the relative population of individual assignations. **c** Distribution of differences between *Inter*- and *Intra*-subunit distances. Median values were calculated from kernel density estimates [20]. **d** Distribution of differences between calculated SASDs and EUCs. Median values were calculated from kernel density estimates [20]

Our results show that using SASDs yields a smaller fraction of *Ambiguous* assignations (11%) than using EUCs (23%) (Fig. 2a). Consequently, the fractions of unambiguous assignations are increased: the fraction of *Intra*-subunit assignations is increased by 5% from 67 to 72% and the fraction of *Inter*-subunit assignations is increased by 7% from 10 to 17%. On the other hand, more than half (53%) of residue pairs could be assigned neither as *Intra-* nor as *Inter*-subunit (Fig. 2a, label *Non-acc.*). A large fraction of Non-accessible assignations can be explained by the fact that SASDs are longer than EUCs and thus more likely above than the threshold. The sum of median EUC and a median increase of distance when SASDs are calculated for *intra*-subunit and *inter*-subunit distances are 33.64 Å and 35.37 Å, respectively (Additional file 1: Figure S1) – that is comparable to our threshold of 33 Å.

To test if the rise of the distances that are above the threshold is also the main reason for the reduction of the number of ambiguous assignations with SASD, we investigated if the same effect can be obtained when lowering the threshold to 30, 27 and 25 Å when using EUCs. The fraction of *Ambiguous* assignation did indeed decrease with reducing threshold (Fig. 2b, Additional file 1: Figure S2). While thresholds of 30 and 27 Å still yielded a higher fraction of *Ambiguous* assignations than using SASDs (17 and 14%, respectively), the same result of 11% could be obtained when using a threshold of 25 Å. However, reducing threshold did not increase the fraction of *Inter*-subunit assignations as it did in the case of SASDs. Furthermore, the majority of initial ambiguous assignations were assigned as *Ambiguous* or *Intra*-subunit, while they were almost equally redistributed between *Ambiguous* (5%), *Intra*-subunit (5%) and *Inter*-subunit (4%) when using SASDs (Fig. 2a). This suggests that using SASDs has a somewhat smaller overall effect on the increase of *Inter*-subunit distances than it does on the increase of *Intra*-subunit distances. To confirm this assumption, we compared differences between *Inter*- and *Intra*-subunit distances in *Ambiguous* assignations using both methods. Results show that these differences are normally distributed when calculating EUCs with a median difference of 4.48 Å. However, the distribution is slightly skewed towards smaller differences when using SASDs (*inter*-subunit distances are less often longer than *intra-subunit*; Fig. 2c). The smaller overall effect on *Inter*-subunit distances is also evident if we compare the increase of *Intra*- and *Inter*-subunit distance when SASDs are used over EUCs (Fig. 2d). The median difference is 11.79 Å in case of *Intra*-subunit distances, compared to a median increase of 8.64 Å in case of *Inter*-subunit distances.

Our analyses, taken together, clearly show that using SASDs instead of EUCs provides significant improvement in reducing the ambiguity of XL-MS data assignation based on the calculated *inter*-residue distances alone. Additionally, using SASDs results in a higher number of identified *Inter*-subunit connections, which are used as spatial restraints in the modeling of homo-oligomeric protein complexes.

### Considering the *Intra*-subunit alternative improves the results of modeling of homo-oligomeric protein complexes

Even though our results show that using SASDs instead of Euclidean distances can reduce the ambiguity of cross-link assignation, some cross-links will remain ambiguous. We investigated the effect of the scoring function to score ambiguous cross-links as *Inter-* or *Intra-*subunit on the precision of modeling results.

Ideally, the performance of different scoring approaches would be assessed on a representative dataset of homo-oligomeric protein complexes with different stoichiometries. However, modeling of higher-than-dimer order homo-oligomers introduces another level of ambiguity as each identified *inter*-residue connection also has multiple ambiguous *Inter*-subunit alternatives. To simplify the comparison, we focused on symmetric homo-dimers only, as they are also by far most predominate oligomeric state within homo-oligomers [3].

We performed our analysis using a representative dataset of 41 homo-dimers. XL-MS data was simulated by extracting lysine residue pairs with inter-residue SASDs below the threshold (33 Å) and used to guide protein-protein docking by assigning each potential cross-link a Matched and Non-accessible cross-link (MNXL) [18] score. MNXL score distinguishes between cross-links that have inter-residue distance below the threshold (matched) and those which don’t (non-accessible). We then used different scoring functions to calculate the total *inter*-subunit MNXL score of a model and compared the highest-ranking models with the initial structure of homo-dimer to assess scoring function’s precision (for more details see Materials and Methods).

First, we examined if treating ambiguous cross-links as ambiguous instead of designating them as *Inter*-subunit to obtain the maximal number of spatial restraints, is indeed important. To do so, we created the following scoring functions:

- the first one scored all ambiguous cross-links as *Inter*-subunit and was oblivious to the existence of *Intra*-subunit alternative (*Oblivious*);
- the second one scored *Inter*-subunit alternative but did not penalize non-accessible *Inter*-subunit cross-links, if *Intra*-subunit alternative was matched *(Normal*).

Because scoring algorithms usually address cross-links as oriented (i.e., a cross-link between subunits A-B is not the same as a cross-link between subunits B-A, which is otherwise the case in homo-oligomers), two variations of scoring functions were used:

- one that addressed cross-links as oriented and thus evaluated alternatives A-B and B-A separately (*oriented*) and
- one that considered only the alternative with the highest MNXL score (*stringent*).

**Fig. 3:**
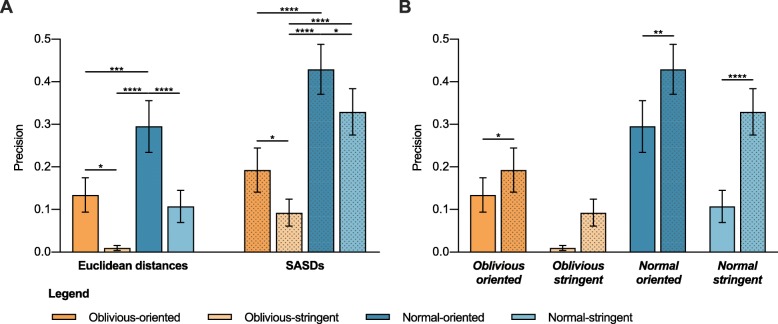
Evaluation of the effect of addressing cross-links as ambiguous (*Oblivious* vs. *Normal*) and oriented (*oriented* vs. *stringent*) on docking precision. **a** Comparison of precision of *Oblivious-oriented*, *Oblivious-stringent*, *Normal-oriented,* and *Normal-stringent* scoring functions. **b** Comparison of average precision when using SASDs over EUCS for all evaluated scoring functions. Results obtained with SASDs are presented as dotted bars. Values represent the mean precision of all structures in the dataset with SEM. Quade ranking test, followed by Quade’s post-hoc test for unreplicated blocked data with Bonferroni correction * *p*-values < 0.05, ** 0.01, *** 0.001, **** 0.0001

Results of the comparison show that *Normal* scoring functions have higher precision than their *Oblivious* counterparts with both methods used for distance calculation (SASD and EUC) (Fig. 3a, Additional file 2: Table S2)). Even tough *Oblivious* scoring functions use more spatial restraints per model, penalizing non-accessible *Inter*-subunit connections, when *Intra*-subunit counterpart is matched, greatly reduces the precision. This demonstrates that *Intra*- and *Inter*-subunit alternatives should always be considered as a single restraint and not scored individually.

Surprisingly, evaluating *Inter*-subunit alternatives individually improves the precision of protein-protein docking, as *oriented* scoring functions have much higher precision than *stringent* scoring functions (Fig. 3a).

With regards to the method of choice for inter-residue distance calculation, SASDs give better results compared to EUCs (Fig. 3b), although the difference is not statistically significant when using Oblivious-oriented scoring function. This supports our previous findings that SASDs are better at resolving the ambiguity of *Intra*- vs. *Inter*-subunit assignation.

### Only the cross-links that cannot be matched as *Intra*-subunit should be considered as *Inter*-subunit restraints

After establishing that *Intra*-subunit alternative should be considered when *Inter*-subunit alternative is non-accessible, we examined in which case a cross-link should be scored as *Inter*-subunit (with regards to the inter-residue distances) if both alternatives are possible. We considered three options:

- option *All* scored all cross-links as *Inter*-subunit, even if the *Intra*-subunit option was matched and had a higher score;
- option *Only best*-scored cross-links as *Inter*-subunit only if the *Inter*-subunit score was higher than the *Intra*-subunit;
- option *Non-Intra* had the strictest criterium and scored cross-links as *Inter*-subunit only if the *Intra*-subunit alternative was non-accessible.

These three options were applied to *Normal*-oriented and *Normal-stringent* scoring functions introduced above.

**Fig. 4:**
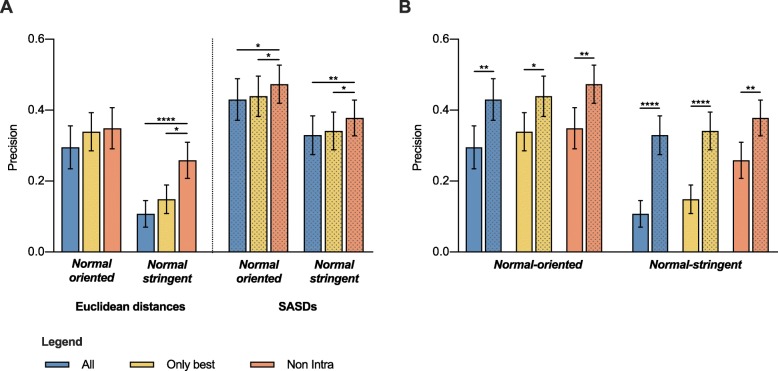
Evaluation of the effect of different approaches to scoring ambiguous cross-links as *Inter*-subunit on docking precision. **a** Comparison of the precision of options All, *Only best* and *Non-Intra*. **b** Comparison of average precision when using SASDs over EUCS for all evaluated scoring functions. Results obtained with SASDs are presented as dotted bars. Values represent the mean precision of all structures in the dataset with SEM. Quade ranking test, followed by Quade’s post-hoc test for unreplicated blocked data with Bonferroni correction * *p*-values < 0.05, ** 0.01, *** 0.001, **** 0.0001

Results suggest that the more conservative scoring function is better. The *Non*-*Intra* option gives the highest precision in all analyzed scoring functions, followed by *Only best* and *All*, which gives the lowest precision (Fig. 4a). The difference between *Non*-*Intra* and *All* is statistically significant in all cases, except when using *Normal*-*oriented* scoring functions and calculating EUCs. On the other hand, the difference between *Only best* and *All* is not statistically significant, even though the average precision of the former is higher in all cases.

In agreement with our previous findings, *Normal*-*oriented* always yields better results than *Normal*-stringent scoring function (Fig. 4a) and using SASDs outperforms EUCs (Fig. 4b).

### Imposing symmetry improves modeling performance

It has been already reported that cross-linking data can be used to predict if a protein complex is symmetric or not, based on the identified interaction interfaces [7]. We investigated the effect of imposing symmetry to spatial restraints in the scoring function on modeling performance.

In a perfectly symmetric homo-oligomer – most homo-oligomers are symmetric – the distance between residue pair A-B is identical to its counterpart B-A. Distribution of distance differences of these pairs in our homo-dimer dataset shows that this is indeed the case. More than 95% of EUCs differences are below 1 Å (Additional file 1: Figure S2). Differences of SASDs are a bit more broadly distributed, but still smaller than 5 Å in more than 90% (Additional file 1: Figure S3).

Two ways of imposing symmetry were considered:

- Scoring *Inter*-subunit alternative as matched only if both *Inter*-subunit distances were matched (*Symmetry-matched*) and
- Scoring *Inter*-subunit alternative as matched if at least one *Inter*-subunit distance is matched and the difference between *Inter*-subunit distances is smaller than 5 Å. (*Symmetry-difference*).

These two scoring functions were compared to *Normal-oriented* and *Normal-stringent* for all three options discussed above (*All, Only best, Non-Intra*).

**Fig. 5:**
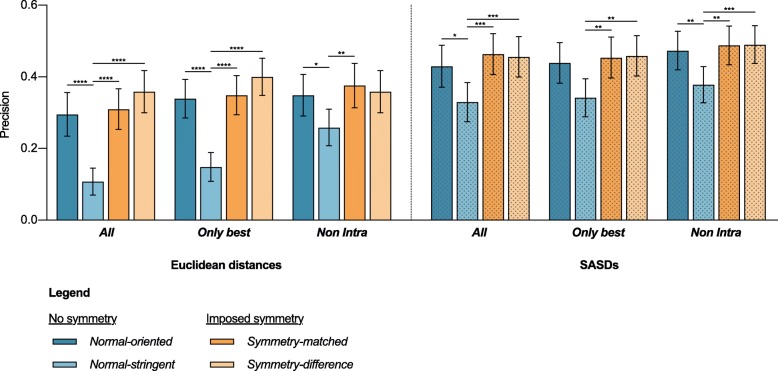
Evaluation of the effect of imposing symmetry on docking precision. Comparison of docking precision with or without directly imposed symmetry. Results obtained with SASDs are presented as dotted bars. Values represent the mean precision of all structures in the dataset with SEM. Quade ranking test, followed by Quade’s post-hoc test for unreplicated blocked data with Bonferroni correction *p-values < 0.05, ** 0.01, *** 0.001, **** 0.0001

Both symmetry imposing scoring functions performed better than both *Normal* scoring functions in all cases (Fig. 5, Additional file 1: Table S1). Conversely, the differences between *Normal-oriented*, *Symmetry-matched* and *Symmetry*-*difference* were not statistically significant.

We believe the reason for this comparable performance of *Normal-oriented* scoring function is that considering cross-links as oriented and scoring both alternatives separately, doubles the score for each cross-link, when both *Inter*-subunit alternatives are matched. This also favors symmetric models and thus gives comparable results to *Symmetry-matched* and *Symmetry-difference* scoring functions, which favorize symmetry by design. However, it needs to be noted that both *Symmetry* scoring functions evaluate the symmetry by comparing the distances of both *Inter*-subunit alternatives, while *Normal-oriented* scoring function gains its increase in performance by over scoring the experimental data.

### Summary: using SASDs with stringent conditions and imposing symmetry gives the best results

Based on our comparisons we conclude that choice of method for inter-residue distance calculation, a decision on when to score ambiguous cross-links as *Inter*-subunit and implying the symmetry in the scoring function itself has an important effect on docking performance.

With regards to the method of inter-residue distance calculation, our analyses clearly show SASDs should be used instead of EUCs.

Ambiguous cross-links should be considered as such as seen from a comparison of *Normal* and *Oblivious* scoring functions. *Inter*-subunit alternatives should be scored only when *Intra*-subunit alternative is non-accessible – option *Non-Intra* has the highest precision of the three options analyzed (*All, Only Best*, *Non-Intra*).

Including symmetry also improves docking precision, whether symmetry is imposed in the scoring function itself by comparing the distances of *Inter*-subunit alternatives or favorized as a consequence of improperly addressing cross-linking as oriented and scoring both *Inter*-subunit alternatives separately.

## Discussion

Many proteins self-assemble to form homo-oligomers, comprised of two or more identical subunits. Structural characterization of homo-oligomeric protein complexes is crucial for the understanding of their role in biological processes. While homo-oligomers have some inherent characteristics such as symmetry, which can be exploited in the process of structure determination, the fact that they are comprised of identical building blocks also presents some additional challenges. In case of modeling protein complexes based on spatial restraints obtained from chemical cross-linking, the obtained linkages cannot be unambiguously assigned as *intra*- or *inter*-subunit without labeling of subunits with heavy isotopes, which is not applicable to all investigations [12, 13]. Here we present a comprehensive evaluation of different methods for inter-residue distance calculation and different scoring approaches for resolving this ambiguity based on data analysis alone.

Our results show that using the appropriate methods for inter-residue distance calculations, calculating SASDs instead of EUCs, substantially reduces the number of ambiguous assignations (Fig. 2a), while providing more *inter*-subunit assignations. Calculating SASDs instead of EUCs generally has a larger effect on *Intra*-subunit than on *Inter*-subunit distances (Fig. 2c-d), probably because a direct path (EUC) between two residues on the same subunit is more likely to cross the protein occupied space than when residues are located on different subunits. As distances that cross the protein occupied space cannot represent the actual connection between the residues via the cross-linker, SASDs therefore also produce spatial restraints that are representing the actual experimental information more adequately. The benefit of using SASDs is also evident from our evaluation of how different scoring approaches effect protein-protein docking precision, as scoring functions employing SASDs consistently perform better than those employing EUCs (Figs. 2, 3 and 4). The superiority of SASDs is in agreement with previous evaluations of methods for distance calculations in the modeling of proteins and protein complexes, comprised of different subunits [18].

Even though SASDs can reduce the extent of ambiguity, cross-links that cannot be assigned unambiguously remain. In the literature, there are several different approaches to consider these ambiguous assignations as *inter*-subunit spatial restraints when modeling homo-oligomeric protein complexes. The most conservative approach is only to consider those, which can be unambiguously assigned based on overlapping peptide sequences while discarding the rest [21, 22]. Other approaches include assignation of cross-links based on the band of origin in SDS-PAGE analysis [23, 24], considering only those that can’t be matched without considering additional subunit [25, 26], or assigning cross-links to the alternative, which has a more favorable distance [8, 27]. As the first two approaches are based on experimental information additional to the identified connected residues, we focused our evaluation of scoring functions based only on distance information. Results of our comparison reveal that the most conservative option of scoring cross-links as *inter*-subunit only, when the *intra*-subunit alternative cannot be matched, gives the best results (Fig. 4a). Even though other options provide a higher total number of restraints, the fact that some of them are falsely assigned as *inter*-subunit seems to be detrimental for the success of homo-oligomer modeling. While our comparisons were made exclusively on dimers, we believe the underlying principles of our findings can be applied to higher-order homo-oligomers, where several *inter*-subunit alternatives exist.

Similarly to the findings of others [7, 28], we showed that symmetry could be exploited to improve modeling results (Fig. 5). Both of our symmetry imposing scoring functions *Symmetry-matched* and *Symmetry-difference* score *inter*-subunit alternative only when both *inter*-subunit alternatives (A-B and B-A) are sufficiently similar. The underlying reasoning is related to the recently proposed contact-base symmetry score (CBS) [28], except that CBS evaluates symmetry on the level of the whole protein complex, while we only consider it on the level of individual cross-links. It is surprising, however, that similar precision can be obtained by incorrectly addressing the cross-links as oriented, thus scoring each *inter*-subunit alternative separately.

Further evaluation would be required to determine if this would also hold in a real experiment when not all cross-links are detected, and cross-links that do not correspond to the actual structure (false positives) are also present in the dataset. Unfortunately, we were not able to find enough experimental cross-links dataset of C2-symmetric homo-dimers with known 3D structures. We compared our findings obtained from simulated cross-links with four experimental datasets (PDB: 1F05, 1IRI, 2PSN, 4MZV) and with a bootstrapped dataset with only 1, 5, 10 and 20% coverage (Additional file 1: Figure S4-S7). While both results from the experimental dataset as well as results from the simulated lower coverage seem to generally agree with our conclusions based on simulated cross-links, the number of evaluated protein complexes is far too small to enable any reliable evaluation.

## Conclusions

We showed that proper evaluation of chemical cross-linking-based spatial restraints in the modeling of homo-oligomeric protein complexes can improve the precision of modeling prediction. Modeling algorithms, which employ chemical cross-linking as a type of spatial restraints, should at a minimum address the ambiguity of intra- vs. inter-subunit assignation, when models are scored, instead of addressing each alternative individually. Such optimizations will facilitate investigations of homo-oligomeric protein complexes and in turn, increase our understanding of their structure and function.

## Methods

### Comparison of methods for inter-residue distance calculations

3D structures of homo-oligomers were downloaded from 3D Complex database [29] (version 6, based on PDB database on March 1st, 2015). We included only the structures with at most 90% amino acid sequence similarity (QS90), high resolution (2.5 Å or lower) and at least two subunits, to exclude monomers. Structures, which were found to have incorrect stoichiometry (annotations “YES” and “PROBYES” in PiQSi database [30]) were discarded. During our analysis, we also excluded proteins with multiple structures with different oligomeric states, structures with split polypeptide chains and structures, for which *inter*-residue distances could not be calculated within three days. This yielded a final dataset of 13,110 structures.

EUCs and SASDs between lysine residues were calculated with Jwalk [18] (v 1.3). EUC is calculated as the distance of a straight line between their C_α_-atoms, without considering their solvent accessibility. Jwalk calculates SASD by placing the proteins on a grid, calculating the solvent accessible surface and using a Breadth-First Search to elucidate the shortest distance.

In its original version, Jwalk does not address the *intra*- vs. *inter*-subunit ambiguity as each *inter*-residue connection is defined with residue number and chain id. For our research, we modified the algorithm to treat all connections between the same residue numbers as a group of connections with all available combinations of chain ids. To avoid calculating redundant distances due to symmetry, only distances between lysine pairs, which had at least one endpoint residue located within the first polypeptide chain in the structure were calculated. If there were more *Inter*-subunit alternatives, only the shortest one was saved. For comparison of assignations, only residue pairs, which had *intra*- or/and *inter*-subunit alternative matched (EUC equal or less than the threshold of 33 Å) and had both *intra*- and *inter*- alternative calculated, were considered (1,151,199 residue pairs). These potential cross-links were then assigned as *intra*-subunit (*Intra*), *inter*-subunit (*Inter*), ambiguous (*Ambiguous*) or non-matched (*Non-accessible*) based on the *intra*- and *inter*-subunit distances. Briefly, a cross-link was assigned as *Intra* or *Inter* if only one of those alternatives had distance ≤33 Å, Ambiguous if both distances were ≤ 33 Å and *Non-accessible* if none of the distances was ≤33 Å (or endpoint residues were not solvent accessible in case of SASDs).

Figures were prepared with Matplotlib graphics package [31] and Seaborn visualization package [20].

### Scoring function comparison dataset

The experimental cross-linking data of homo-oligomers is scarce. To ensure the results of our scoring function comparisons are generally applicable, we used simulated data. First, we extracted models from the 3D Complex database [29] that matched the following criteria: at most 30% amino acid sequence similarity (QS30), high resolution (2.5 Å or lower), structure is a C2 symmetric dimer and stoichiometry was found correct during manual inspections (annotations “NO” in PiQSi database [30]). Second, proteins with multiple structures with different oligomeric states, structures with split polypeptide chains and structures without any simulated cross-links were removed, yielding the dataset of 47 protein structures. Six protein structures which had less than ten models with C_α_-RMSD from the recreated initial structure ≤10 Å (see below) were excluded from the final dataset. The final dataset thus had 41 protein structures.

### Generation of models and calculation of *inter*-residue distances

For each protein structure in the dataset, we generated C2-Symmetric and random homo-dimer models using SymmDock [32, 33] and PatchDock [33, 34], respectively. One thousand models with the highest SOAP score [35] from each algorithm were extracted for analysis. In some cases, when PatchDock produced less than 1000 models, all generated models were taken.

Initial structures were also recreated by superimposing the second subunit with the copy of the first one, to ensure the initial structure, had exactly the same chains as models. The recreated initial structures were then used to simulate the cross-linking data. We calculated SASDs between all lysine residues using Jwalk [18] (v 1.3) and extracted a list of residue pairs with *intra*- or *inter*-subunit SASD equal or below the threshold (33 Å). These residue pairs were considered as simulated cross-linking data, and their EUCs and SASDs were calculated in all models using Jwalk [18] (v 1.3).

### Scoring functions

We used Matched and Non-accessible cross-link (MNXL) [18] scoring function as the basis:

$$ MNXL\ score\ \left[ distance\right]=\left\{\begin{array}{c}N\left(18.62,35.94\right)\ \\ {}-0.1\end{array}\begin{array}{c} if\ distance\le 33\ {\AA}\\ {} else\end{array}\right. $$

The MNXL score assigns each cross-link a positive value if the distance is under 33 Å (matched). The exact value is calculated based on the probability given by normal distribution with mean (18.62) and variance (35.94) calculated from experimental cross-link database [18]. If the distance is greater than 33 Å – or if any of the endpoint residues is not solvent accessible in case of SASDs – a penalty of −0.1 is assigned (non-accessible).

To enable comparison, the same parameters were used for EUCs, SASDs, *intra*-subunit, and *inter*-subunit distances. Models were ranked by total MNXL score (sum of MNXL scores of *inter-subunit* cross-links). We created several scoring functions that had different criteria when to include a single *inter*-subunit MNXL score in the *inter*-subunit MNXL score:

- Oblivious**:** *inter*-subunit MNXL score is included regardless of the value of its *intra*-subunit alternative.
- Normal: if *inter*-subunit MNXL score is non-accessible, the penalty is not assigned if the *intra*-subunit alternative is matched but rather scored with a neutral value of 0.0.
- Oriented: both *inter*-subunit alternatives (A-B and B-A) are scored separately.
- Stringent: only the *inter*-subunit alternative with the higher MNXL score included.
- All: *inter*-subunit MNXL score is included even if the *intra*-subunit alternative had a higher MNXL score.
- Only best: *inter*-subunit MNXL score is included only if it is higher than if the *intra*-subunit alternative.
- Non-intra: *inter*-subunit MNXL score is included only if the *intra*-subunit alternative is non-accessible.
- Symmetry matched: *inter*-subunit MNXL score is included only if both *inter*-subunit alternatives are matched.
- Symmetry-difference: *inter*-subunit MNXL score is included if at least one of *inter*-subunit alternatives is matched and if the difference in their distances is smaller than 5 Å.

The performance of scoring functions was compared in terms of precision, calculated as the percentage of near-native models in top-10 models with highest total *inter*-subunit MNXL. A model was considered a near-native if the C_α_-RMSD from the recreated initial structure was ≤10 Å.

### Comparison of simulated cross-links with experimental data

We used experimental cross-linking data for four C2-symmetric homo-dimers that had high-resolution 3D structures available (PDB: 1F05, 1IRI, 2PSN, 4MZV) [8, 36, 37]. For these four protein complexes, models were generated, scored, and evaluated in the same manner as described above for the scoring function comparison dataset. For comparison, the analysis was also performed with all possible cross-links, defined as in the scoring function comparison dataset analysis and with simulated 1, 5, 10 and 20% coverage (number of obtained cross-links vs. number of all possible cross-links).

To ensure simulated coverage was representative, 1000 random datasets were bootstrapped for each percentage value [18].

### Statistics

Results of scoring function performance are represented as mean precision ± SEM. Graphs were drawn with GraphPad Prism version 8.0.0 for Mac (GraphPad Software, San Diego, California USA). Statistical comparison was made with Quade ranking test [38] (STAC python library [39]) followed by Quade’s post-hoc test for unreplicated blocked data with Bonferroni correction [38, 40, 41] (scikit-posthocs, v 0.4.0, https://pypi.org/project/scikit-posthocs/).

## Additional files

Additional file 1: **Figure S1.** Inter-residue distance distributions an *Intra*- and *Inter*-subunit EUCs distributions. b Distribution distance increases when using SASDs over EUCs for *Intra*- and *Inter*-subunit alternatives. Median values were calculated from kernel density estimates [20]. **Figure S2**. **a-c** Effect of threshold distance on assignation when using EUCs. The total values obtained with each threshold are shown in header row and column, while the body of the heatmap shows the proportions of combinations. Values in parentheses represent the relative proportion of assignations obtained using a lower threshold, excluding *Non-accessible*. In row headers, *Amb.* stands for *Ambiguous* and *Non-acc.* Stands for *Non-accessible*. **Figure S3.** Distribution of distance differences between both *Inter*-subunit alternatives in recreated initial structures**. Figure S4.** Comparison of simulated cross-links with experimental data (PDB: 1F05). For each scoring function, the precision obtained with experimental cross-links, all possible cross-links and average precision obtained from bootstrapped dataset at 1, 5, 10, and 20%. a Comparison of *Oblivious* and *Normal* scoring functions. b Comparison of scoring options *All, Only best* and *Non-Intra* applied *to* Normal-oriented and *Normal-stringent* scoring functions. c Comparison of symmetry imposing scoring functions *Symmetry-matched* and *Symmetry-difference* with *Normal* scoring functions. **Figure S5.** Comparison of simulated cross-links with experimental data (PDB: 1IRI). For each scoring function, the precision obtained with experimental cross-links, all possible cross-links and average precision obtained from bootstrapped dataset at 1, 5, 10, and 20%. a Comparison of *Oblivious* and *Normal* scoring functions. b Comparison of scoring options *All, Only best* and *Non-Intra* applied *to* Normal-oriented and *Normal-stringent* scoring functions. c Comparison of symmetry imposing scoring functions *Symmetry-matched* and *Symmetry-difference* with *Normal* scoring functions. **Figure S6.** Comparison of simulated cross-links with experimental data (PDB: 2PSN). For each scoring function, the precision obtained with experimental cross-links, all possible cross-links and average precision obtained from bootstrapped dataset at 1, 5, 10, and 20%. a Comparison of *Oblivious* and *Normal* scoring functions. b Comparison of scoring options *All, Only best* and *Non-Intra* applied *to* Normal-oriented and *Normal-stringent* scoring functions. c Comparison of symmetry imposing scoring functions *Symmetry-matched* and *Symmetry-difference* with *Normal* scoring functions. **Figure S7.** Comparison of simulated cross-links with experimental data (PDB: 4MZV). For each scoring function, the precision obtained with experimental cross-links, all possible cross-links and average precision obtained from bootstrapped dataset at 1, 5, 10, and 20%. a Comparison of *Oblivious* and *Normal* scoring functions. b Comparison of scoring options *All, Only best* and *Non-Intra* applied *to* Normal-oriented and *Normal-stringent* scoring functions. c Comparison of symmetry imposing scoring functions *Symmetry-matched* and *Symmetry-difference* with *Normal* scoring functions. **Table S1**. Comparison of average precisions of scoring functions *Normal-oriented, Normal-stringent, Symmetry-matched,* and *Symmetry-difference*. (PDF 366 kb)

Additional file 2: **Table S2.** Results of Scoring function comparisons. Results are presented as the precision of each scoring function for homo-oligomeric protein complex in the analyzed dataset. Precision of both PathDock an Symmdock withouth cross-linking-based distance restrains is also presented. (XLSX 16 kb)

## Data Availability

The dataset used and/or analyzed in this study are available from the corresponding author upon reasonable request.

## Abbreviations

EUC: Euclidean distance
MNXL: Matched and Non-accessible cross-link
SASD: Solvent accessible surface distance
XL-MS: chemical cross-linking coupled with mass spectrometry
