# Supplementary material for: Proper evaluation of chemical cross-linking-based spatial restraints improves the precision of modeling homo-oligomeric protein complexes
Source: BMC Bioinformatics. 2019 Sep 9;20:464. doi: 10.1186/s12859-019-3032-x (PMC6734309; doi:10.1186/s12859-019-3032-x)
Supplement: Supplementary file 1 — Figure S1. Inter-residue distance distributions an Intra- and Inter-subunit EUCs distributions. b Distribution distance increases when using SASDs over EUCs for Intra- and Inter-subunit alternatives. Median values were calculated from kernel density estimates [20]. Figure S2. a-c Effect of threshold distance on assignation when using EUCs. The total values obtained with each threshold are shown in header row and column, while the body of the heatmap shows the proportions of combinations. Values in parentheses represent the relative proportion of assignations obtained using a lower threshold, excluding Non-accessible. In row headers, Amb. stands for Ambiguous and Non-acc. Stands for Non-accessible. Figure S3. Distribution of distance differences between both Inter-subunit alternatives in recreated initial structures. Figure S4. Comparison of simulated cross-links with experimental data (PDB: 1F05). For each scoring function, the precision obtained with experimental cross-links, all possible cross-links and average precision obtained from bootstrapped dataset at 1, 5, 10, and 20%. a Comparison of Oblivious and Normal scoring functions. b Comparison of scoring options All, Only best and Non-Intra applied to Normal-oriented and Normal-stringent scoring functions. c Comparison of symmetry imposing scoring functions Symmetry-matched and Symmetry-difference with Normal scoring functions. Figure S5. Comparison of simulated cross-links with experimental data (PDB: 1IRI). For each scoring function, the precision obtained with experimental cross-links, all possible cross-links and average precision obtained from bootstrapped dataset at 1, 5, 10, and 20%. a Comparison of Oblivious and Normal scoring functions. b Comparison of scoring options All, Only best and Non-Intra applied to Normal-oriented and Normal-stringent scoring functions. c Comparison of symmetry imposing scoring functions Symmetry-matched and Symmetry-difference with Normal scoring functions. Figure S6. Compariso [file 12859_2019_3032_MOESM1_ESM.pdf]

Additional File 1

Proper evaluation of chemical cross-linking-based spatial restrains improves precision of modeling homo-oligomeric protein complexes

Aljaž Gaber, Gregor Gunčar, Miha Pavšič
*Correspondence should be addressed to M.P. (miha.pavsic@fkkt.uni-lj.si)

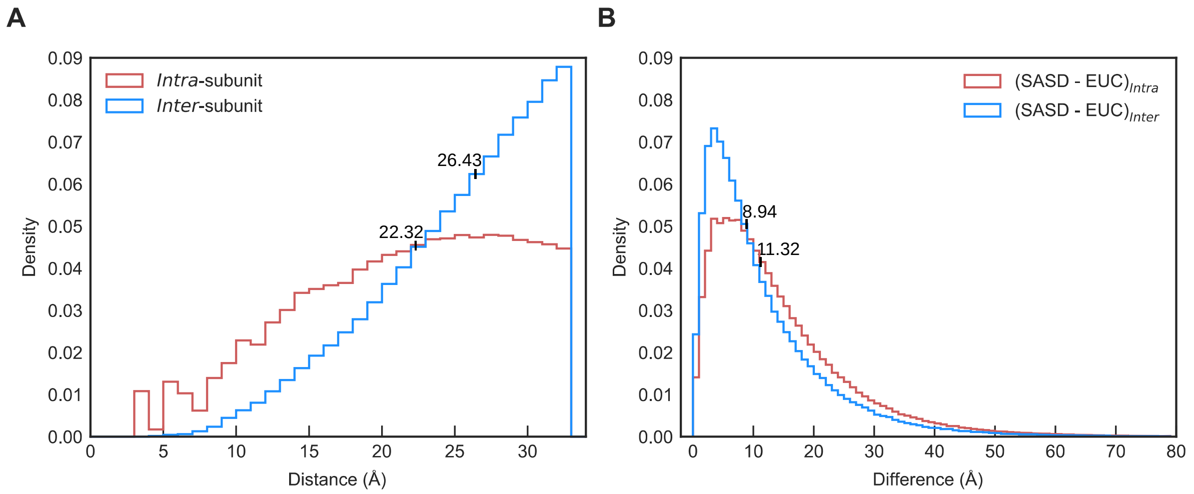

Supplementary Figure 1: Inter-residue distance distributions. A: *Intra*- and *Inter*-subunit EUCs distributions. B: Distribution distance increases when using SASDs over EUCs for *Intra*- and *Inter*-subunit alternatives. Median values were calculated from kernel density estimates [20].

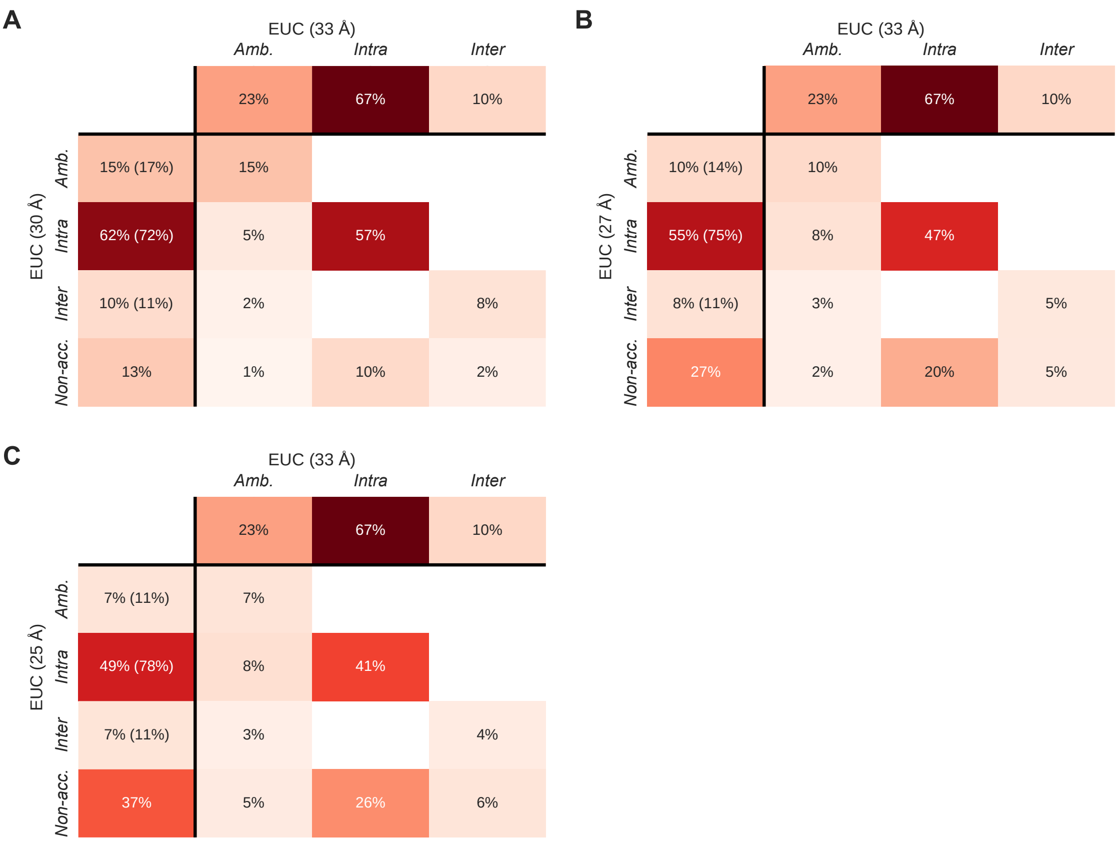

Supplementary Figure 2. a-c Effect of threshold distance on assignation when using EUCs. The total values obtained with each threshold are shown in header row and column, while the body of the heatmap shows the proportions of combinations. Values in parentheses represent the relative proportion of assignations obtained using lower threshold, excluding *Non-accessible*. In row headers, *Amb.* stands for *Ambiguous* and *Non-acc.* stands for *Non-accessible*.

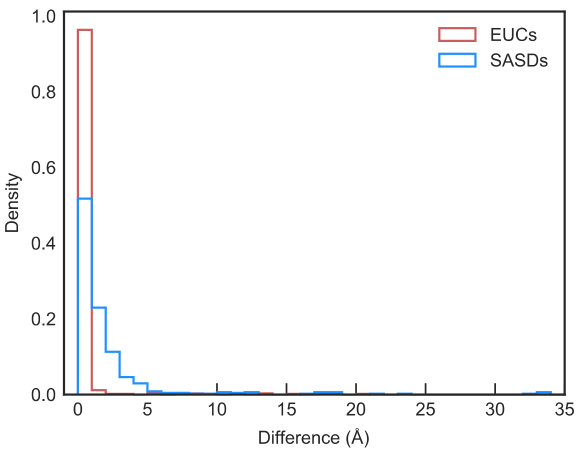

Supplementary Figure 3: Distribution of distance differences between both *Inter*-subunit alternatives in recreated initial structures

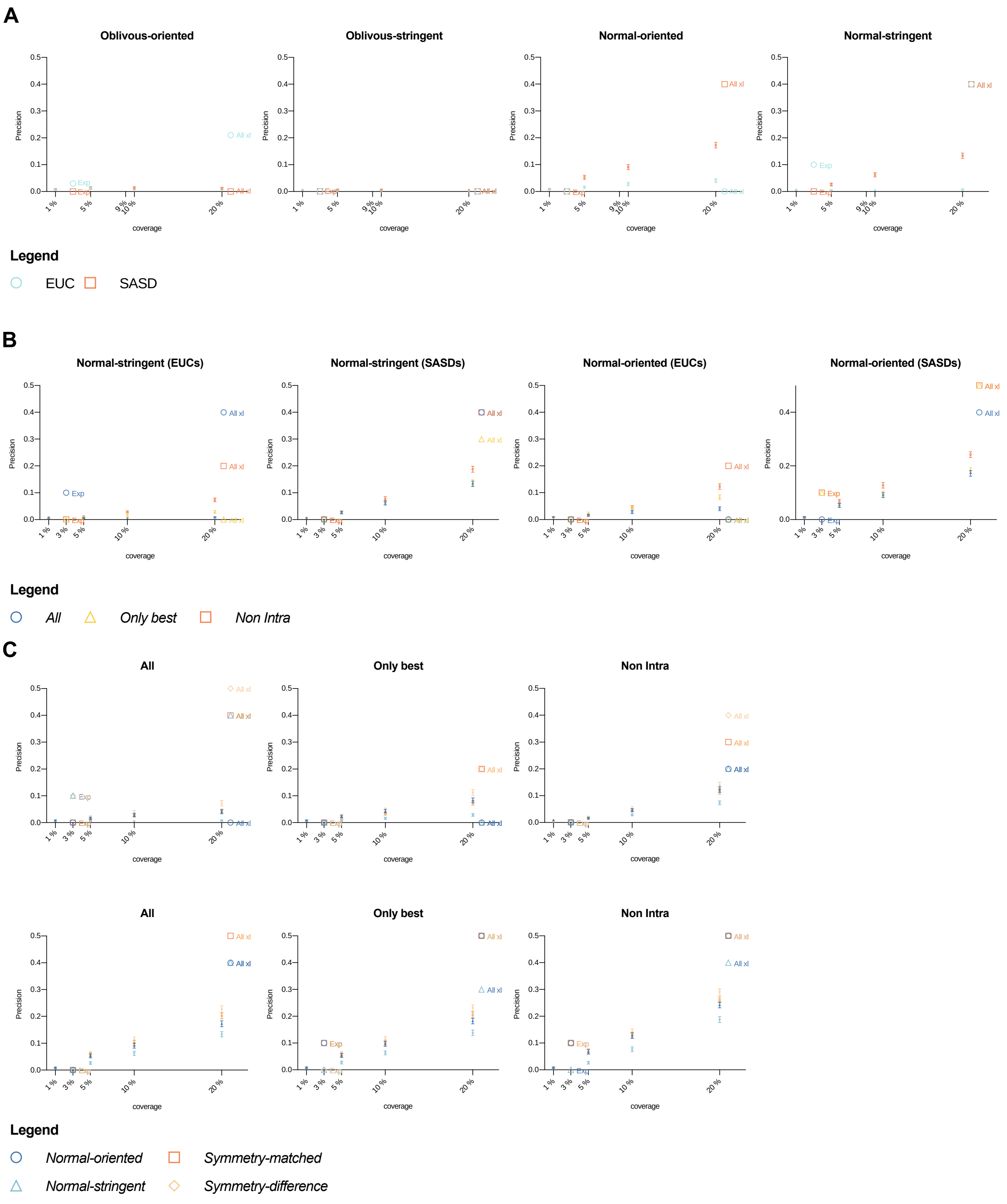

Supplementary Figure 4: Comparison of simulated cross-links with experimental data (PDB: 1F05). For each scoring function, precision obtained with experimental cross-links, all possible cross-links and average precision obtained from bootstraped dataset at 1%, 5%, 10% and 20%. **a** Comparison of *Oblivious* and *Normal* scoring functions. **b** Comparison of scoring options *All, Only best* and *Non Intra* applied *to* Normal-oriented and *Normal-stringent* scoring functions. **c** Comparison of symmetry imposing scoring functions *Symmetry-matched* and *Symmetry-difference* with *Normal* scoring functions.

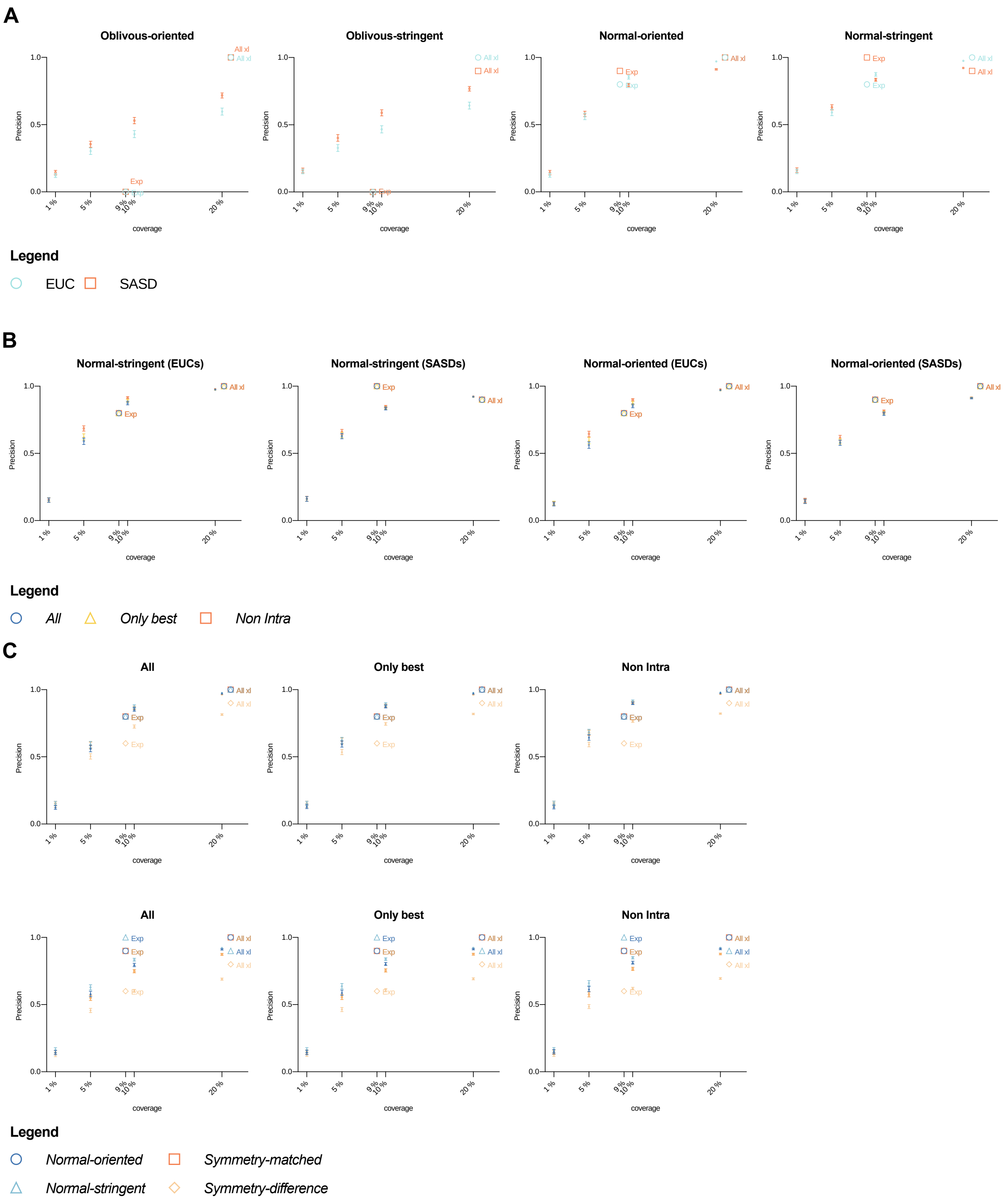

Supplementary Figure 5: Comparison of simulated cross-links with experimental data (PDB: 1IRI). For each scoring function, precision obtained with experimental cross-links, all possible cross-links and average precision obtained from bootstraped dataset at 1%, 5%, 10% and 20%. **a** Comparison of *Oblivious* and *Normal* scoring functions. **b** Comparison of scoring options *All, Only best* and *Non Intra* applied *to* Normal-oriented and *Normal-stringent* scoring functions. **c** Comparison of symmetry imposing scoring functions *Symmetry-matched* and *Symmetry-difference* with *Normal* scoring functions.

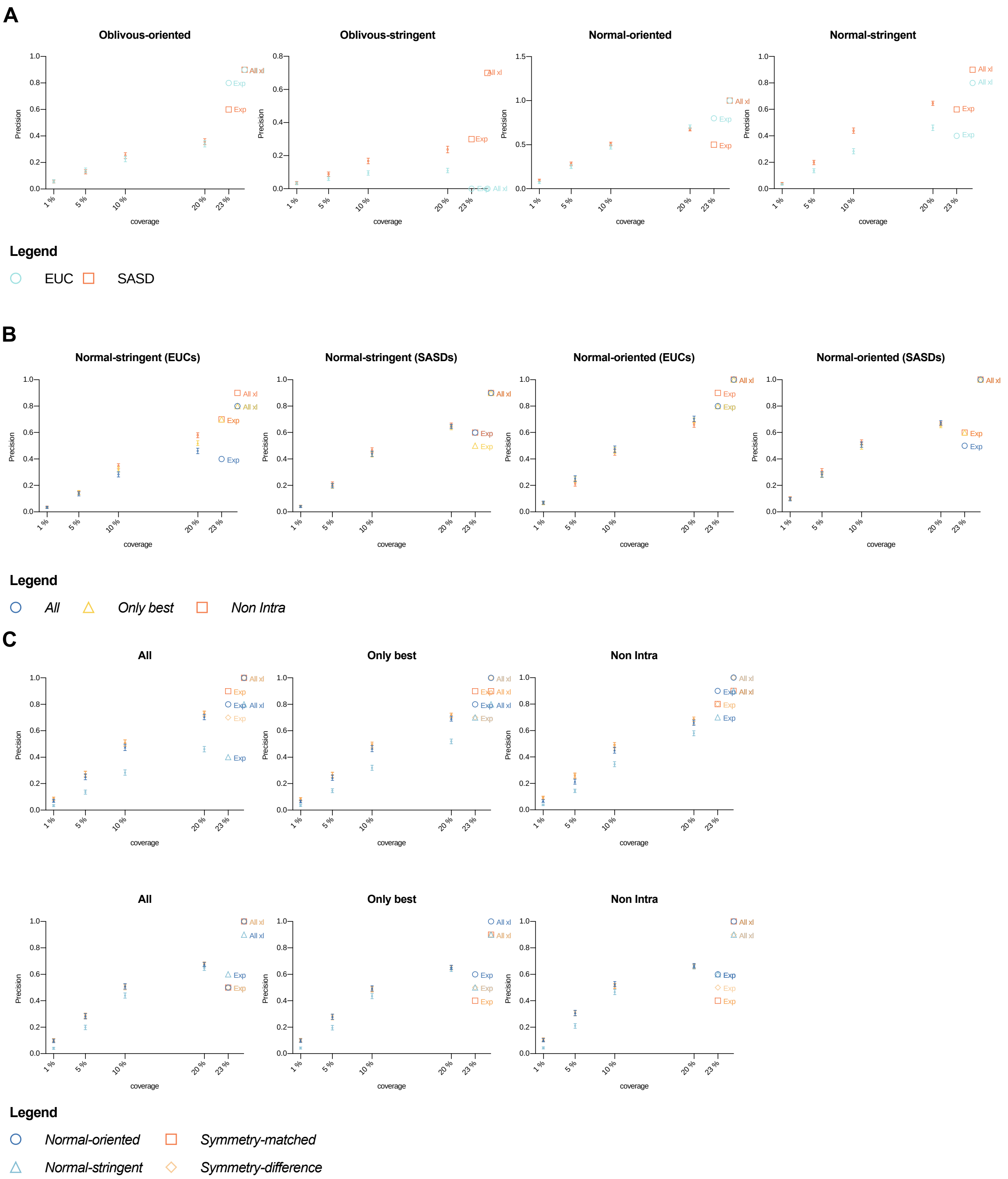

Supplementary Figure 6: Comparison of simulated cross-links with experimental data (PDB: 2PSN). For each scoring function, precision obtained with experimental cross-links, all possible cross-links and average precision obtained from bootstraped dataset at 1%, 5%, 10% and 20%. **a** Comparison of *Oblivious* and *Normal* scoring functions. **b** Comparison of scoring options *All, Only best* and *Non Intra* applied *to* Normal-oriented and *Normal-stringent* scoring functions. **c** Comparison of symmetry imposing scoring functions *Symmetry-matched* and *Symmetry-difference* with *Normal* scoring functions.

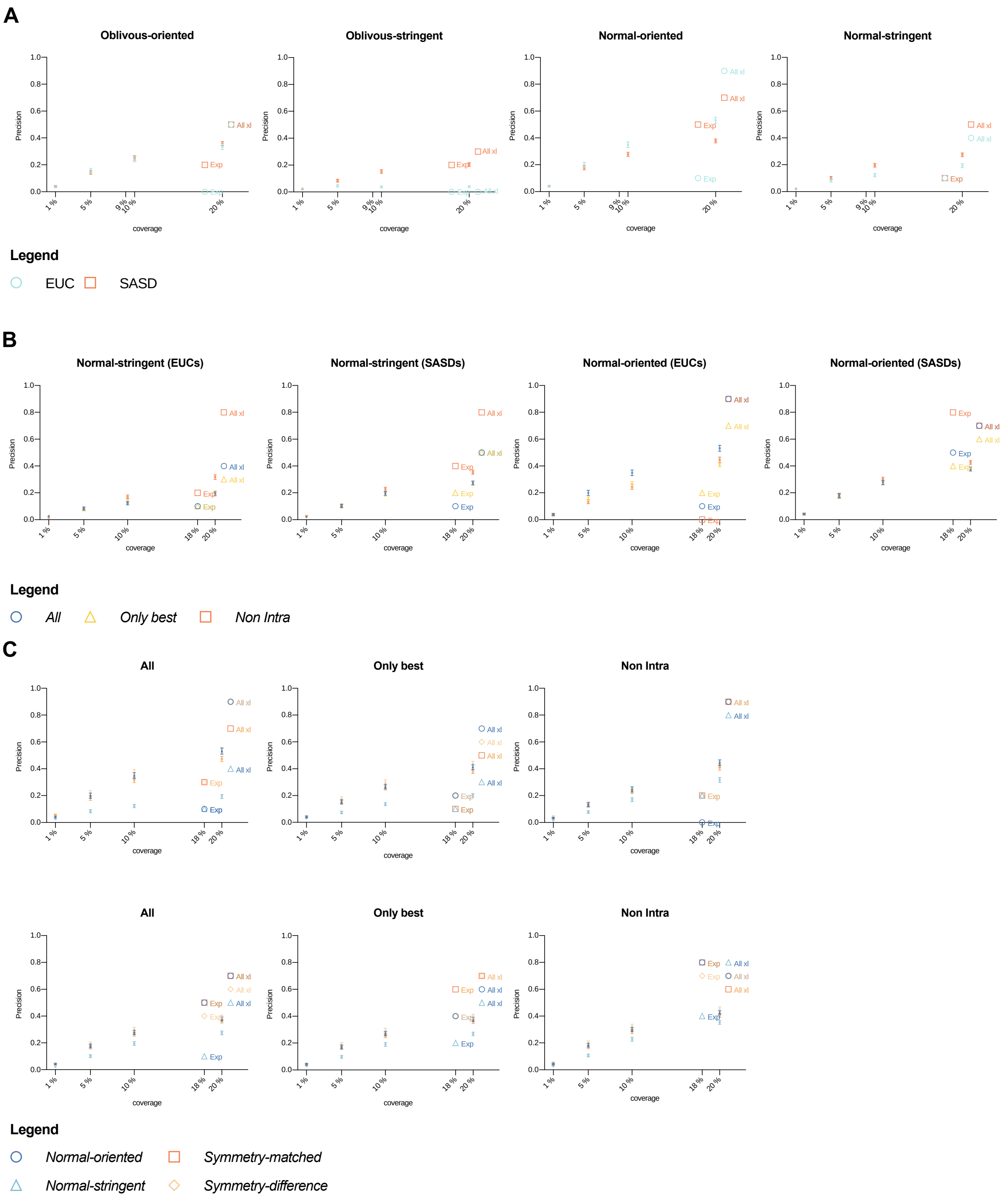

Supplementary Figure 7: Comparison of simulated cross-links with experimental data (PDB: 4MZV). For each scoring function, precision obtained with experimental cross-links, all possible cross-links and average precision obtained from bootstraped dataset at 1%, 5%, 10% and 20%. **a** Comparison of *Oblivious* and *Normal* scoring functions. **b** Comparison of scoring options *All, Only best* and *Non Intra* applied *to* Normal-oriented and *Normal-stringent* scoring functions. **c** Comparison of symmetry imposing scoring functions *Symmetry-matched* and *Symmetry-difference* with *Normal* scoring functions.

	Euclidean distances			SASDs		
	All	*Only best*	*Non Intra*	*All*	*Only best*	*Non Intra*
Normal-oriented	0,295	0,339	0,349	0,429	0,439	0,473
Normal-stringent	0,107	0,149	0,259	0,329	0,341	0,378
Symmetry-matched	0,310	0,349	0,376	0,463	0,454	0,488
Symmetry-difference	0,359	0,400	0,359	0,456	0,459	0,490

**Average precision:**	Highest			Lowest

Supplementary Table 1: Comparison of average precisions of scoring functions *Normal-oriented, Normal-stringent, Symmetry-matched* and *Symmetry-difference*.
